# Supplementary figures and images for: Systems Level Analyses Reveal Multiple Regulatory Activities of CodY Controlling Metabolism, Motility and Virulence in Listeria monocytogenes
Source: PLoS Genet. 2016 Feb 19;12(2):e1005870. doi: 10.1371/journal.pgen.1005870 (PMC4760761; doi:10.1371/journal.pgen.1005870)

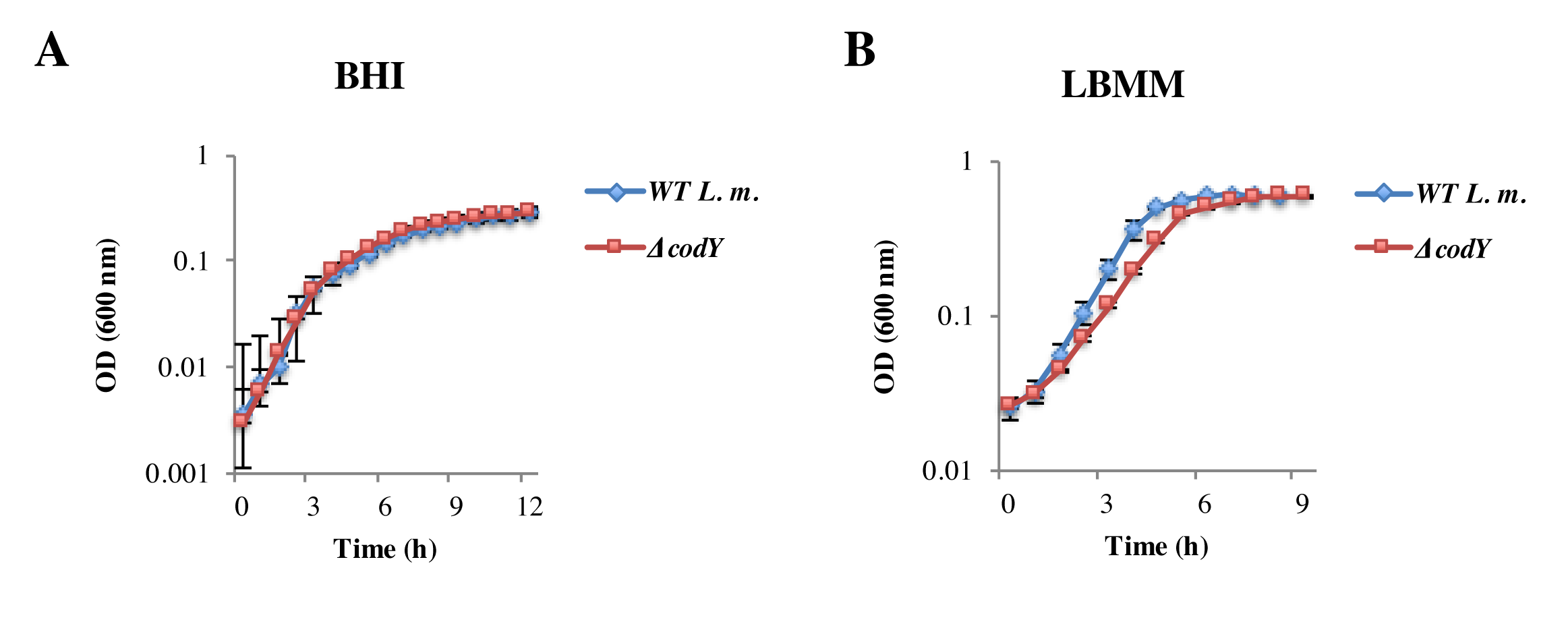

Supplement: S1 Fig — Optical density measurements of WT L. monocytogenes and ΔcodY bacteria during growth in BHI (A) and LBMM (B). Results are average of 3 independent experiments. Error bars represent standard deviation. (TIF) [file pgen.1005870.s001.tif]

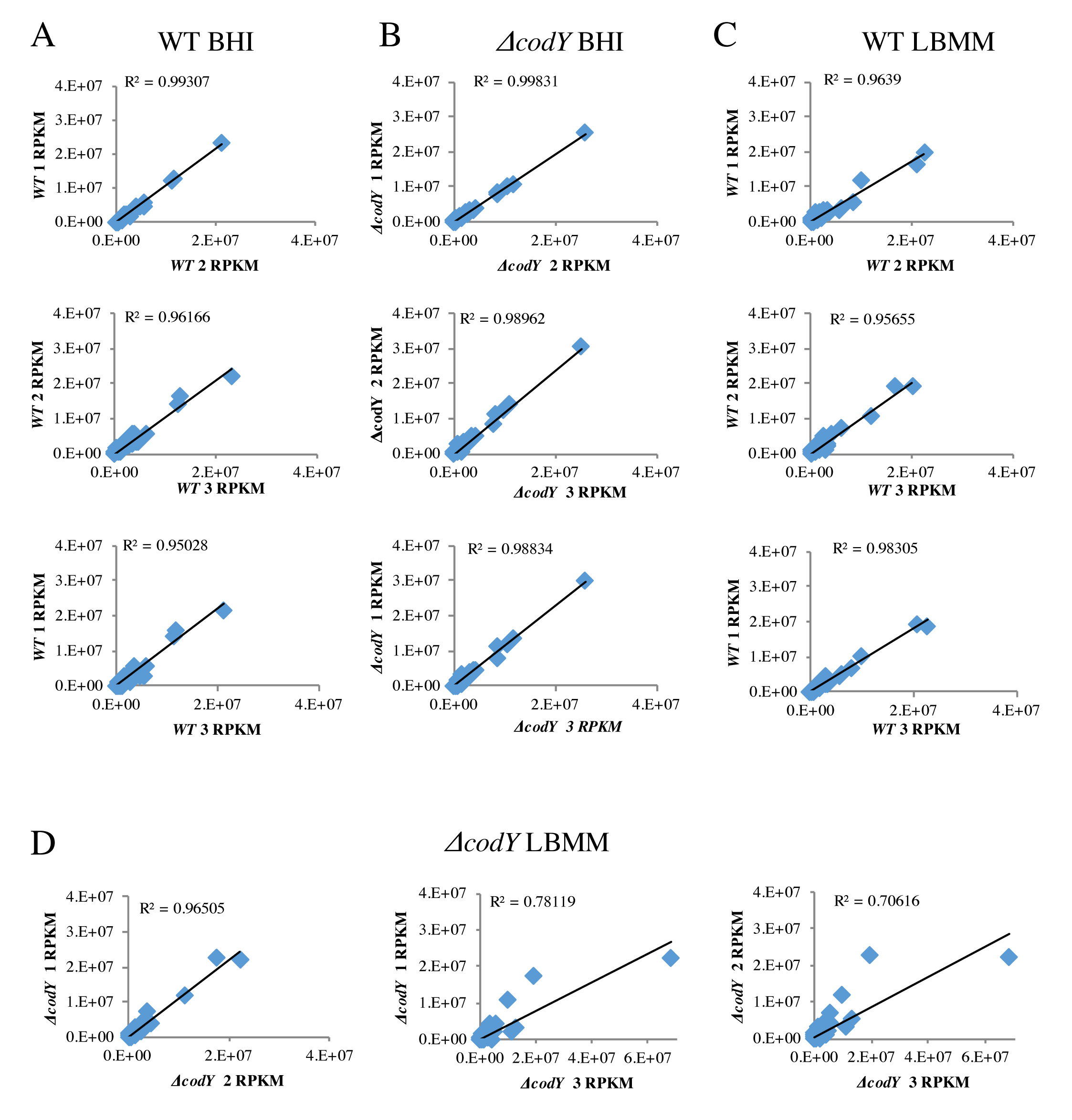

Supplement: S2 Fig — Correlations between biological repeats of WT (A) and ΔcodY (B) L. monocytogenes transcriptome analyses in BHI medium. Correlations between biological repeats of WT (C) and ΔcodY (D) L. monocytogenes transcriptome analyses in LBMM medium. Each point in the graphs represents a gene and each axis represents a biological repeat. R2 represents linear regression correlation values. Of note, ΔcodY 3 LBMM sample was found to have more then twice of its reads aligning to non-coding regions compared to all other RNA-Seq samples. Furthermore, its correlation with the two other ΔcodY LBMM samples was significantly low. Therefore, this sample was omitted from the analysis. (TIF) [file pgen.1005870.s002.tif]

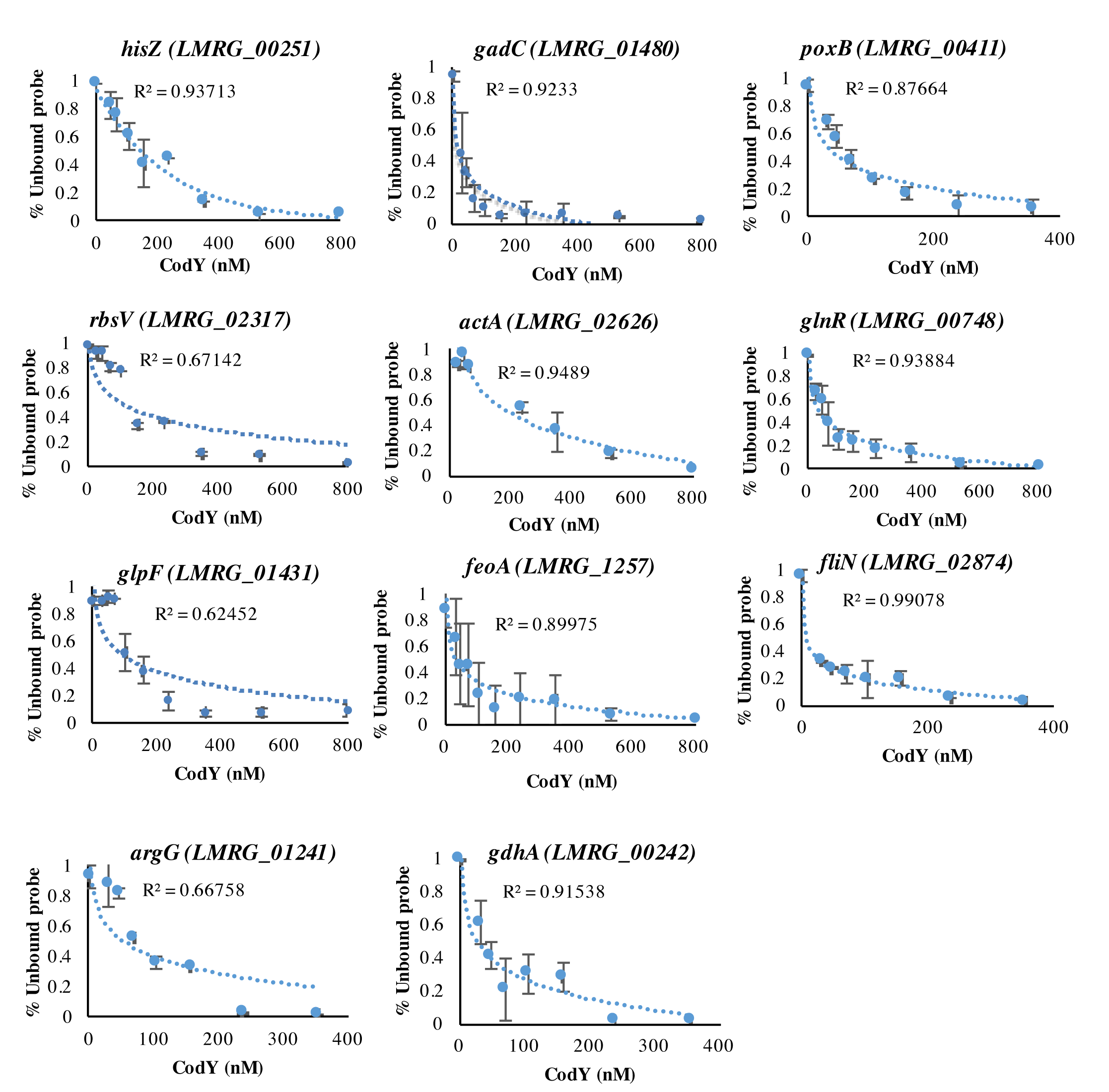

Supplement: S4 Fig — An averaged regression analysis is shown for each probe. Analysis is based on densitometry measurements of the ratio between the free DNA probe and the total DNA probe at each CodY concentration (i.e., each lane) using ImageJ software [86]. Quantifications of 2–3 biological repeats were fitted via exponential least-squares regression analysis. The average apparent KD values depicted in the manuscript are based on 2–3 independent regression analyses made for each probe and are not derived from the averaged graphs presented here. Error bars represent standard error of the mean. (TIF) [file pgen.1005870.s004.tif]
