# Supplementary material for: Systems Level Analyses Reveal Multiple Regulatory Activities of CodY Controlling Metabolism, Motility and Virulence in Listeria monocytogenes
Source: PLoS Genet. 2016 Feb 19;12(2):e1005870. doi: 10.1371/journal.pgen.1005870 (PMC4760761; doi:10.1371/journal.pgen.1005870)
Supplement: S3 Fig — Schematic representation of the regulatory regions of hisZ (A), rbsV (B), glpF (C), argG (D), gadC (E), feoA (F), actA (G), gdhA (H), poxB (I), glnR (J) and fliN (K) genes, which were selected for EMSA analysis. The regulatory region of each gene is presented. CodY motifs are highlighted. (PDF) [file pgen.1005870.s003.pdf]

A

hisZ (LMRG_00251)

```
                                                                                                              >-35
                                                                                               >CodY Box 5 mm
ATTCAACCCTCTTTCAAAATTCTTTTTCCCAAGTGCTTTAAATGGTACACTAATTATACCATAAGCAACTATTTTTCTATTCATTATTTACAAAAACGCT  < 100

      >-10
TTAACGTGCTAATATGCTAATAAACTAAAATATCGTAAAGGAGCTAACCAGATGAACTTAAATAAGAACTTACCAACTGGAACACGTGATAAACTTTTTC  < 200

>LMRG_00251
GTGAAGCACGAGCTGCATACAAAATTGAGCAACAAGTGAATCATTATTTT  < 250
```

B

rbsV (LMRG_02317) first gene in *sigB* operon

```
                                                                                 >-35
GAAGAAGTGAAGACGTACCTCATTCTTTAGAGAGCATTTACTGGTGAGTAAACGTATTTAAAGAGTGGAGCGTTTTTATTTTTGAATGTTTTAATTTTAT  < 100

      >-10            >CodY Box 4 mm
TAGTTAGGGTAAAATCGACAGTATACTTAAAATTAGATGGGGTGAAGTGATGAATATTAGTATAGAAATAAAAGAACGTGATACTGACCACATAGACATA  < 200

            >LMRG_02317 [*]
TTTGTTGCTGGGGAGATCGATGCTTATACAGCGCCAAAGGTAAAAGAAGCATTAGAAGTATATCAAGTTAAAGAGGG  < 277
```

C

glpF (LMRG_01431)

```
                                                              >-10
                                      >-35                  >CodY Box 4 mm
AACCGTCACTTGTGGCGGTTTTTTTGTGTTTTCATCTTTGTGAATTAATTTTGATTTCTTTTAAAATTGGTTATAACTCTCGCGAATTGGGGTAAAAGTA  < 100

AAGAGGCAGATGAAGATACATTGTGAAAAATTGATATGATAGGACAAGTCGTTTGATTGACAGCGTTTTCTTGATATGATTAAATAGACTCAAGTTAATT  < 200

AACATGTCAGGAGAAGAGATGACAATCGAAACGATGCAAGGGTACTAGTGTGCCTACGTTTTGATTGTCATTCTTTTTTTGAAGTTAATTGATAATCGGA  < 300

ATGTAAGGGGACTAGTAAGTGTTAAAATTCCGGTGCTACAAATAAGCAAGAAGAAGGAGTTGGAAAAGATGATTGACACAAGTTTAGCAACACAATTTCT  < 400

                              >LMRG_01431
AGGTGAAGTCATTGGTACGGCTATACTAATTATTTTAGGTGCTGGTGTCGTAGCAGGTGTTTCACTGAAAAGATCCAAAGCAGAGAATG  < 489
```

D

argG (LMRG_01241)

```
ATTCGCTCAACTCCTTCAATATAACTTCAATAACAAGTATAGCAGTTTTATGAACAGGAACCAATTTATTACTATTTGAAATGATTATTTTTATTTAGAA  < 100

                       >CodY Box 5 mm          >-35                    >-10
ATGTATTTTTAAGCATAATTATGAATAAATGAAAATAAAAAGGTTGATATCTAAAAGCATCGATGTTAATATAAATACAAATGTTTGTATATTTATGAAT  < 200

                                                                       >LMRG_01241
AGGGGATGTTTAAAATGGCGAAAGAAAAAATCGTATTAGCTTACTCAGGTGGGTTAGATACTTCTGTGGCAATTCAGTGGTTAGTAGAATCAGGTTATGA  < 300

AGTTATTGCATGTT  < 314
```

gadC (LMRG_01480)

E

```
                                            >-35                    >-10
CATTAAATCCTCCCTCTTCCCATTATTACCTTTCAATATCCTTAATGGACATTTAACATCTATATACAATACACCCATTTTTTTTAAAGTGCAAGAGTTT  < 100

TTTACTAAAAATGGAAAAAAATTTAATGAATGCGTTTGCTGCGAATAGAACAACTATAAGCATTGTCTCAAAAATTTTTCCTGGCTAAGAAAAAAACTGG  < 200

          >CodY Box 4 mm
AATAATTTTCGAAAACAAACGTGCTAATCTTATACATGTTCCAACAAACAATTACTTAGGAGGTGCTTTTCTCGTGTTAACTGATGATGTTCCAAAAAAA  < 300

TCACATTTACCATTAATTAAGCATCATTCTAATTACTTATAGCTTCGTTTAGTTGTATTTTCGCAAACAAAAAATTGGGAGGTAACATACAATGTCTAAA  < 400

                                          >gadC (LMRG_01480)
CAAGCAAAGTCTTTAACCTTATTCGGATTTTTCGCCATCACGGCTTCCATGGTTATGACCGTTTATGAATATCCAACATTTGCCACTTCCG  < 491
```

F

feoA (LMRG_01257)

```
GTTAATTGCCTCCATTAATATACATATTATAGTTACCATTTTACTATAAAAAAGAACAACTTGACAGAAAAAAAGTGATTAAAAACAGCTCCAGCACTTA  < 100

TTTCCGTGTATCAATACAGAGGTAAAAAGGCATGCCACTTAGGCAAAAGACGGCTATGCTCTTAAGAAATTTTTTTGCTGTTATACCCTACTATTTTGCT  < 200

                            >-35
                 >CodY Box 5 mm                      >-10
ATTCTAACAGCAATTTCAAAAAAACACTTCATATTAAGGTCTGTTCGTGATAAAATGAACATAGTTGATAATGATTATCATGTTCATTACATAACATAAA  < 300

                           >CodY Box 5 mm                                                      >LMRG_01257
AGGAGGCCTGCTAGCTTATGCAATTAAATGAAACTGCTGTCGGTGAAAAAGTTCGCATCTCAGAATTAAAAATTGAGAACGCTATGCTTAAACGTCGCTT  < 400

ACTTGCACTTGGTTGCGATGAAGGTTGTGATATTTGTATCAAACAAAAAGGACTATTTGGTG  < 462
```

G

actA (LMRG_02626)

```
>-35
>CodY Box 4 mm                              >-10
TTAACAAATGTTAGAGAAAAATTAATTCTCCAAGTGATATTCTTAAAATAATTCATGAATATTTTTTCTTATATTAGCTAATTAAGAAGATAATTAACTG  < 100

>CodY Box 4 mm
CTAATCCAATTTTTAACGGAATAAATTAGTGAAAATGAAGGCCGAATTTTCCTTGTTCTAAAAAGGTTGTATTAGCGTATCACGAGGAGGGAGTATAAGT  < 200

>LMRG_02626
GGGATTAAATAGATTTATGCGTGCGATGATGGTAGTTTTCATTACTGCCAACTGCATTACGATTAACCCCGACATAATATTTGCAGCGACAGATA  < 295
```

H

gdhA (LMRG_00242)

```
>CodY box 3 mm   >-35
CTTCTATATCTACTCATTAAAACCTAAGCAAACGTTCTCATTAACTACATTTTAAAAAATTTCAGAAAAATTGGTTTTTATGCTTGAAAATATTCTCAAC  < 100

>-10
AATGTGTAAACTAACAACATAGGTAAGAAAAGTTGACCAAATTTCAGAAAAATTTACTTAACGCACACAAGCCAATGTTTTTCTTCTTTATAACATTGCA  < 200

TGTATTTGCGTCAGGTTTTCTACTTTTCCTTATAGTCAAAATATTTAAACAAGGAGCGATTATAGTATGGCACAAACATCCACTATCCAAAACGACACAA  < 300

>LMRG_00242
AGGCAGCCGAAGAATACGCAGCTCGAGTGTTTGAAACAATTAAACAACGGAATCCTGGCGAAACGGAATTCCACCAAGCAGTTGAAGAATTCTTAAACTC  < 400

TGTTATCCCAGCCCTTGCAAAAGAACCTAAATACGAAGCAAATGGCATTTTAGAACAGTTAACAGAACCCGAACGCCTTATCAGTTTCCGAGTTCCATGG  < 500

GTGGATGATTCTGGCA  < 516
```

I

poxB (LMRG_00411)

```
>-35   >-10
TATAATTCTCCTTTAATAGTTTAATGTTGGTTAAACTAGCTGAAGGATTGAAGAGTTTATTTCACTCTTACTTTCTAAACAATCCTAAAGCCATTGTTAG  < 100

>CodY box 4 mm
AAAGTCGAGAGCAGTGCAATTTTGTTTGCATCTCTTTGATCACCACCTTGCCTTCATTATAAGCACTTCTGAATTGTTCGTCAATTCTCTTTTATGAATA  < 200

CTCTTCTAAAAACAGGGTAAACGAAAACTATTGGACATTAGAGAAAACATCCATGGGAGGAGAAAGTAAAATGAAAAAAGTAAAAGCAAGTGAAACACTC  < 300

>LMRG_00411
GTACAAACATTAAAAAATTGGGGTATCGACCACGTATATGGCTTGCCCGGCGATTCGATTGACACCGTAGTTGACGCTTTACGAAAAGAAC  < 391
```

J

glnR (LMRG_00748)

>**CodY Box 4 mm**

AAAAGTAACATTT**ATTTTTCAGAAAAAA**TTTTTTAATTTTTACCGCGATGGATTCATTCGCGGTTTTTTTGTATGTAATGCATTGGTATATAAGGCTTTA < 100

>**-35** >**-10**

TCTATAATGTTAGATTATCTAACATAAAT**TTGACA**AAACATCTAACGTA**CATTATAAT**GCTACTATCATCAAAAAAATAATTATGTTTTGAGGGGGAATT < 200

>**LMRG_00748**

GTC**GTGAGCGAAAAGGAAATCCGGAGATCGATGCCGCTTTTTCCAATTGGGCCTGTGATGAAGCTAACTGATCTCACTGCTAGACAAATTCGCTACTATG** < 300

< 300

K

fliN (LMRG_02874)

>**CodY Box 4 mm**

ACAATCACATATCTCTCTATCCTATAGTATTATT**GATTTTCTGTTTATT**TTTATAATAACAATATATGTTAGTTGTGTAAAGGGAACGCGAGTAGGTCAA < 100

AAGGATTGGGTATGAAGAACCTTTTTTTGAGTTAAATAGAAAAATATAAATCGAATAATGAGAAGTTTTCCTTTGATACAGCTGTTACACTGGCTGTTTT < 200

>**-35** >**-10**

TTTATT**TTTACA**CACAATTTCAT**TTTTTAAAA**AAAGTATAGATTTTTTCTGGATTTGGTGTAAAATTTTATATTGCAGTTACAAAAAACACTTTATTGAT < 300

>**CodY Box 4 mm**

ATA**TATTTTGAAAAAATG**ATTTATTTTTGTAATCTGATGAAGGGAGTGAAAGGTGAATTTACCTTACTTGA**ATGGAATATTTTTTGTCACTTTTAAAAAT** < 400

>**LMRG_02874**

TATTGTACTTTTGGCAGTACTTGCGGGAATTTCTTATTTTTTAGTTAAGAAAAACAAGCAAGCGAATCGCACGAGAAAAAGCGAAAATGATCTAATAAAG < 500
